# N95 Filtering Facepiece Respirators during the COVID-19 Pandemic: Basics, Types, and Shortage Solutions

**DOI:** 10.5704/MOJ.2007.002

**Published:** 2020-07

**Authors:** S Srinivasan, WCG Peh

**Affiliations:** Department of Diagnostic Radiology, Khoo Teck Puat Hospital, Singapore

**Keywords:** coronavirus disease, COVID-19, filtering facepiece (FFP) respirator, N95 respirator, personal protective equipment (PPE)

## Abstract

The coronavirus disease 2019 (COVID-19) is highly infectious, with the current pandemic causing significant morbidity and mortality worldwide. As large numbers of frontline healthcare workers (HCWs) have also been infected and several have died, there is much global concern about protective measures for them, particularly those performing surgery or other procedures with close patient contact. Since the beginning of the pandemic, there has been and there remains a shortage in the supply of personal protective equipment (PPE), including the N95 filtering facepiece (FFP) respirator, for HCWs. N95 respirators have filtration efficiency of 95% of aerosol particles. Surgical N95 respirators are used where fluid resistance is also required together with respiratory protection, e.g. during surgery or interventional procedures. The shortage of N95 respirators may be overcome by extended use and reuse - comprising rotation and decontamination by approved techniques. The additional role of powered air-purifying respirators (PAPR) is also discussed.

## Introduction

The current coronavirus disease 2019 (COVID-19) pandemic can be traced to its first reported case in Wuhan, China in December 2019. At the time of writing this manuscript, more than 4.2 million people have been infected worldwide, with deaths occurring in more than 287,000^1^. The incubation period is relatively long (approximately 14 days) and the virus is highly contagious. The spread of virus occurs through airborne droplets and surface contact^2^. Shortage of personal protective equipment (PPE) is a major contributing factor to a large number of healthcare workers (HCWs) contracting COVID-19, in the course of diagnosing, treating and caring for both asymptomatic subjects and patients who are COVID-19 positive^3^. Several hundreds of HCWs have died of COVID-19 worldwide. According to a report released by International Council of Nurses on 6th May 2020, at least 90,000 HCWs have been infected and more than 260 nurses have died^4^.

To prevent spread of airborne respiratory infection such as COVID-19, wearing of masks or filtering facepiece (FFP) respirators are advised. The masks can be simple cloth masks (which can be worn by members of public or outside the high risk areas), surgical masks which are loose-fitting disposable devices that prevent entry of large size droplets that may contain micro-organisms, and N95 FFP respirators. The most important component of PPE for HCWs during this pandemic is the N95 FFP respirator. The U.S. Centers for Disease Control and Prevention (CDC) does not recommend N95 FFP respirators for general public use, stating that they should be reserved for HCWs. In this article, we discuss N95 FFP respirators: types, proper procedure for use, solutions for addressing the current shortage, and disadvantages. The additional role of powered air-purifying respirators (PAPR) is briefly discussed.

## N95 FFP Respirators

The N95 FFP respirators (N95 respirators for short) are approved for healthcare and industrial use by the U.S. National Institute of Occupational Safety and Health (NIOSH). This organisation tests and approves respirators, based on filtration efficiency and performance. N95 respirator filters 95% of all (non-oil) aerosolised particles, hence the name N95. Aerosol, in this context, refers to the suspension of fine solid or liquid particles in air. The higher-grade N100 respirators have a filtration efficiency of 99-100% and are not widely used in healthcare. The European standard, as per the European Committee for Standardization (CEN, French: Comité Européen de Normalisation), classifies FFP respirators into FFP1, FFP2 and FFP3 based on the filtration efficiencies (80%, 90% and 99%, respectively). N95 respirators are equivalent to FFP25.

The N95 respirators are usually tested against uncharged sodium chloride particles measuring 0.1 to 0.3 micrometres in size. The respirator provides 95% protection and permits air flow of around 85 litres per minute^6^. The main use of N95 respirators is to protect HCWs from airborne respiratory infectious diseases such as COVID-19. The advantages of N95 respirators over surgical masks and simple cloth masks are reduced filter penetration of aerosolised particles and absent face-seal leakage^7-10^. During epidemics and pandemics, the demand for N95 respirators frequently outruns available supply.

## Types of N95 Respirators

N95 respirators are classified based on the size and shape. The N95 respirators are approved by NIOSH and few models of the N95 respirators are approved by U.S. Food and Drug administration (FDA) as well^11, 12^. The FDA-approved N95 respirators are resistant to fluid at a high pressure (range of 120-160mm Hg) as well as airborne particles. The surgical N95 respirator is the one approved by FDA and these are highly recommended during surgery or procedures which can cause exposure to high pressure spillage of body fluids (such as arterial bleeding).

**Fig. 1:**
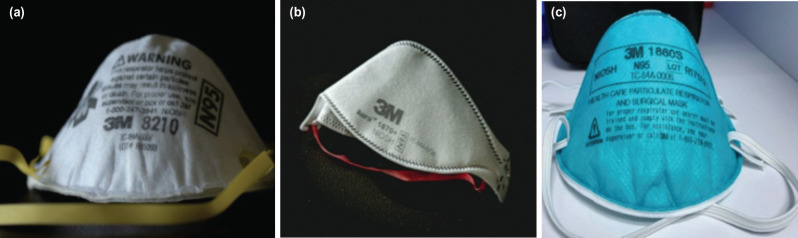
Photographs show some types of N95 respirators. (a) Standard N95 respirator (3M – 8210 model). (b) Surgical N95 respirator (3M-1870 model). (c) Surgical N95 respirator (3M- 1860S model).

The most commonly used N95 respirators are manufactured by 3M [3M, St. Paul, MN, USA]. Some commonly used models are 3M 8210 and 3M 8110 (which are standard N95 respirators); and 3M 1860 and 3M 1870/1870+ (surgical N95 respirators) (Fig. 1). The 3M 1860 model is cup-shaped and model 1870/1870+ is trifold in shape^13^. The surgical N95 respirators made by other manufacturers are Alpha Pro Tech 695 [Alpha Pro Tech, Inc, Salt Lake City, UT, USA], Kimberly-Clark 46727 [Kimberly-Clark, Roswell, GA USA], Gerson 1730 [Louis M. Gerson Co., Middleboro, MA, USA] and Sperian One-Fit HC-NB295F [Sperian Respiratory Protection USA, LLC, Santa Ana, CA, USA]^13^.

The differences between standard N95 and surgical N95 respirators are listed in Table I^14^. The surgical N95 respirators are subset of N95 respirators which are tested for fluid resistance, flammability and biocompatibility. Surgical N95 respirators are the respirators of choice when surgical or interventional procedures are performed on COVID-19 patients, because they have the ability to prevent penetration of high-pressure streams of fluid or blood e.g. injury to vessel during surgery or spurting of blood during arterial puncture. In other circumstances, the standard N95 suffices when only respiratory protection is needed.

**Table I:** Comparison between the standard and surgical N95 respirators

	Standard N95 respirator	Surgical N95 respirator
NIOSH approved	yes	yes
Examples:		
Model number (3M)	3M 8210, 3M 8110	3M 1860, 3M 1870/1870+
FDA approved	no	yes
Protects against airborne particles	yes	yes
(including viruses and bacteria, fumes and dust)		
95% filtration efficiency of aerosol particles	yes	yes
(solid and liquid)		
No latex	yes	yes
Resistant to high pressure release of fluid (120-160mm	Hg) no	yes

## Fit-Test, Seal Check, Donning and Doffing of N95 Respirators

All HCWs should be individually fit-tested for the N95 respirators prior to clinical use. The respirators should form a tight seal without air-leakage, and this is usually checked by experienced and specially-trained infection control personnel, ideally based in the same healthcare facility^3^. Fittest is an objective evaluation to check the tight seal between the face and the facepiece of the respirator. The fit-test should be performed annually or at alternate years. On successfully passing the fit-test, the same model, make and type of N95 respirator should be worn each time by the HCW. If the HCW fails the fit-test due to lack of availability of respirators of suitable size, or has facial asymmetry or facial hair, then the PAPR is an alternative (discussed later in a separate section). Seal check is a procedure carried out by the wearer of a N95 respirator to check air leakage during negative or positive pressure (by forceful inhaling or exhaling)^15^. The seal check is done each time the respirator is worn^3^. The seal check should not be confused with the fittest. The wearing and removing of PPE, including the N95 respirator is called donning and doffing, respectively^16^. The sequence of donning and doffing, along with meticulous hand hygiene, should be strictly followed to avoid contamination.

## Shortage

**Fig. 2:**
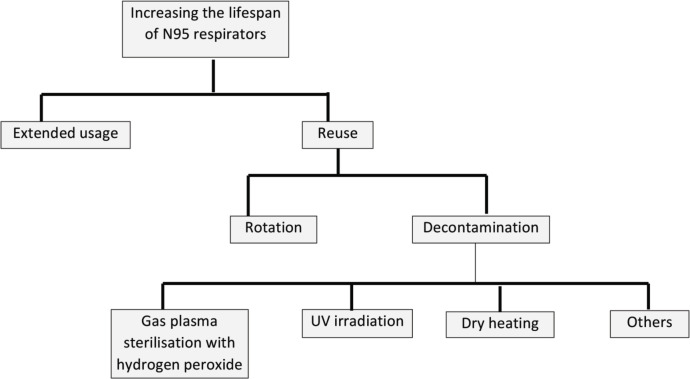
Flow diagram shows summary of methods to preserve the limited supply of N95 respirators.

The COVID-19 pandemic has disrupted supply chains, resulting in a critical shortage of high-demand PPE, including N95 respirators, worldwide. In many countries, HCWs have little choice but to be forced to use substandard PPE and respirators, even home-made ones^17, 18^. Similar situations have also occurred during past pandemics and epidemics^19^. National, local regional and hospital administrative authorities should have the oversight to monitor the demand for PPE, and prioritise the needs of the frontline HCWs who are at the highest risks of exposure to the virus. In the following sections, we discuss some methods which may help to preserve the limited supply of N95 respirators, if needed (Fig. 2).

### Extended use

N95 respirators can be used for up to eight hours without removal. However, there may be slight time duration variations in practice, depending upon hospital or local guidelines. If a transparent shield is worn over the respirator, the life of the respirator can be further extended without contamination or soiling of the respirator surface^12, 19^.

### Reuse

Reuse can be implemented during periods of shortage, if the N95 respirator is worn by the same person, and donning and doffing are carefully performed without touching the inner or outer surfaces of the respirator with bare hands. The CDC recommends reuse up to five times. Reuse can be achieved by rotation of the N95 respirators or by decontamination^20, 21, 22, 23, 24^.

### Rotation

Rotation of N95 respirators is the easiest and most popular technique in many countries, where the supply of N95 respirators is very limited and decontamination facilities are not available. The virus is considered not to be viable after 72 hours, hence it is recommended that the respirator can be used again after 72 hours^2^. Ideally and according to CDC guidelines, at least five N95 respirators are needed for rotation, and strict precautions should be taken during donning and doffing to avoid contamination^12^. The respirators should be preserved in a dry clean cover or a sealed paper bag. The HCW’s name and unique identification number and number of the respirator should be written on the cover or bag label for identification and to avoid confusion. The N95 respirators should not be shared. Seal check should be performed each time it is worn. Each N95 respirator can be reused up to five times. The respirator should be discarded if it fails the seal check, or if it is contaminated, damaged or soiled.

### Decontamination

The techniques used for decontamination should be able to preserve the filtration efficiency of the N95 respirator, maintain the structure and fit of the respirator, and eliminate all the viral or bacterial spore contaminants^18, 19^. Recently, these techniques have also been supported for COVID-1921. Approved techniques consist of hydrogen peroxide gas plasma sterilisation, ultraviolet (UV) irradiation, and use of dry heat, among other methods.

#### Hydrogen peroxide gas plasma sterilisation

Currently, the FDA has granted emergency use authorisation for hydrogen peroxide gas plasma sterilisation by “Sterrad" devices (Advanced Sterilisation Products) [ASP; Irvine, CA, USA] or “Steris" equipment [Steris, Mentor, Ohio, USA]. This is currently the most promising method for effective decontamination and inactivation of COVID-19 virus in used N95 respirators. This technique was initiated by Schwartz *et al* from Duke University^25^. The technique is used to sterilise heat sensitive devices and materials. Vacuum is created within the sterilisation chamber which is then filled with hydrogen peroxide vapour. The hydrogen peroxide builds up to 300-750 parts per million (ppm) for a duration of 20 minutes. At completion of the sterilisation cycle, the de-gassing post-procedure usually takes four hours. There is no residual chemical left as it degrades into water and oxygen^26^. Since vapours are used, there is no risk of toxic material which can remain on the surface of the N95 respirator. The N95 respirators can be safely used after 3-4 sterilisation cycles. However, the fit-test and seal check should be performed before each donning.

#### UV irradiation

Ultraviolet germicidal irradiation (UVGI) has recently been granted approval by the CDC for inactivation of COVID-19 virus in used respirators. In UVGI, the UV rays inactivate the micro-organisms by interfering with the nucleotides in the RNA and DNA, preventing their replication^27^. It was successfully tried at the University of Nebraska Medical Center in Lincoln, Nebraska, USA. The respirators are placed between two towers, with each of them having around eight UV bulbs and the walls of the room are coated with reflective paint^28, 29^. The average time taken for decontamination is 15 minutes. UV treatment technique for decontamination is, however, not widely available.

#### Dry heat

Dry heating can be an effective technique if there are no other options. According to the protocol described by Shroyer^30^, dry heating is performed at 100°C (212 F) for 30 minutes in a pre-heated conventional oven at a processing center. The respirators are placed in a sealed paper bag with name and work location written on it. Recent tests conducted at National Institute of Health (NIH) show that this technique can be effective against COVID-19 virus for two cycles without compromising the quality or the fit of the respirator^23^. This technique has not been approved by CDC.

#### Other techniques

Several other techniques have been described but are still undergoing testing and hence, have not been approved by regulatory authorities for use in the current COVID-19 pandemic. These techniques include moist heating, cleaning with liquid hydrogen peroxide, sodium hypochlorite, alcohol wipes and ethylene oxide. Moist heating is performed at a temperature of 700C with 80-85% relative humidity. However, there is no definite evidence that this is effective to decontaminate COVID-19. The other techniques have not been tested. Microwave heating is not recommended because of the risk of fire.

## Disadvantages of N95 Respirators

Although N95 respirators are considered very safe and effective for HCWs in situations such as the COVID-19 pandemic, there are few disadvantages, namely:

- Requires initial and periodic fit-testing and possibility of being compromised by improper fit (e.g., facial asymmetry, facial hair).
- Cost-effectiveness. N95 respirators of various sizes and types are needed to be stocked in the healthcare facility and individual departments. The supply may be very limited and healthcare facilities in some developing countries cannot afford to stock different types of N95 respirators^31^.
- Poor tolerance due to difficulty in breathing.
- Humidity and moisture build up, leading to deterioration.
- Risk of contamination of areas of face and neck which are not covered by the respirator. In situations where period of exposure is expected to be longer (>30 minutes) in high risk areas, addition of PAPR is recommended.

## Powered Air-Purifying Respirator (PAPR)

**Fig. 3:**
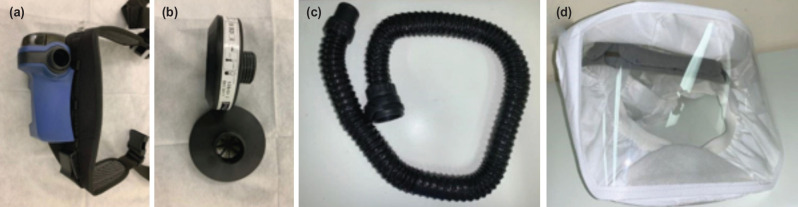
Photograph shows parts of a powered air-purifying respirator (PAPR). (a) Blower unit, (b) filter, (c) connection tubing, and (d) hood.

PAPR is an alternative to the N95 respirator (Fig. 3). It is a battery-powered blower device which enables positive air flow through a filter to a hood or face-piece (covering the HCW’s head and neck). The filters often used are P100 and high-efficiency particulate air (HEPA) filters, which have a filter efficiency of more than 99% and are considered more protective than N95 respirators^31^. PAPR is an additional protective equipment (along with N95 respirator) for HCWs working closely with COVID-19 patients, especially during aerosol-generating procedures, because of higher risk of transmission^32, 33^. PAPRs can be reused but careful cleaning is required after each use. The advantages of PAPR over the N95 respirator include highest level of protection from aerosolised particles, approval as an alternative when fit-test of N95 respirator has failed or difficult to perform, continuous usage and reusability after proper cleaning. Disadvantages include the cost, limited supply, complexity of the multiple parts, time taken for assembly and frequent checks that are needed for the filter (which may be needed to be replaced at regular intervals). The battery is rechargeable and frequent charging of the battery is required. Noise generated by the airflow can be uncomfortable (especially during procedures) and use of stethoscope can be difficult when a PAPR is worn^31^.

## Conclusion

HCWs, especially frontline staff working in high risk areas during the COVID-19 pandemic such as surgeons and other proceduralists, should be familiar with PPE, and N95 respirators in particular. They should be aware of the types and the importance of precise fitting and checks. Extended use and reuse practices can be adopted according to standard guidelines, if there is shortage of supply; but precautions should be taken to avoid self-contamination or cross contamination. PAPR can be used alternatively in circumstances where the N95 respirator is not suitable, or as an additional piece of PPE.
